# Mimivirus Circulation among Wild and Domestic Mammals, Amazon Region, Brazil

**DOI:** 10.3201/eid2003.131050

**Published:** 2014-03

**Authors:** Fábio P. Dornas, Felipe P. Rodrigues, Paulo V.M. Boratto, Lorena C.F. Silva, Paulo C.P. Ferreira, Cláudio A. Bonjardim, Giliane S. Trindade, Erna G. Kroon, Bernard La Scola, Jônatas S. Abrahão

**Affiliations:** Universidade Federal de Minas Gerais, Belo Horizonte, Brazil (F.P. Dornas, F.P. Rodrigues, P.V.M. Boratto, L.C.F. Silva, P.C.P. Ferreira, C.A. Bonjardim, G.S. Trindade, E.G. Kroon, J.S. Abrahão);; URMITE CNRS UMR 6236, IRD 3R198, Aix Marseille Université, Marseille, France (B. La Scola)

**Keywords:** Amazon, mimivirus, megavirus, megavirales, monkey serum, bovine serum, vertebrates, Brazil, viruses

## Abstract

To investigate circulation of mimiviruses in the Amazon Region of Brazil, we surveyed 513 serum samples from domestic and wild mammals. Neutralizing antibodies were detected in 15 sample pools, and mimivirus DNA was detected in 9 pools of serum from capuchin monkeys and in 16 pools of serum from cattle.

The group of nucleocytoplasmic large DNA viruses includes viruses that are able to infect different hosts, such as animals, green algae, and unicellular eukaryotes (*1*). Several members of this group are widely distributed in various environments, actively circulate in nature, and are responsible for outbreaks of medical importance (*2*,*3*). *Mimiviridae*, the newest family in this group, has been researched as a putative pneumonia agent and found in different biomes worldwide (*3*,*5**–**9*). The ubiquity of freeliving amebas and their parasitism by mimiviruses enhances the prospect that diverse environments could shelter these giant viruses (*8**–**10*). Mimiviruses can induce infection in a murine model, have had antibodies detected in patients with pneumonia, and can replicate in murine and human phagocytes (*11**,**12*). Moreover, although some authors suggest that mimivirus is a not frequent pneumonia agent (*4*), mimivirus has been isolated from a human with pneumonia (*3*).

The biomes in Brazil, particularly in the Amazon region, provide the diversity, species richness, and ecologic relationships ideal for identifying circulation of mimiviruses. Preliminary studies found *Acanthamoeba polyphaga mimivirus* (APMV) genomes in samples of bovine serum from Germany (*13*,*14*), indicating that the analysis of samples from vertebrates could be a way to explore and understand the circulation of this group of viruses in nature. We describe the detection of mimivirus antibodies and DNA in 2 mammalian species in the Amazon region of Brazil. 

## The Study

**Figure 1:**
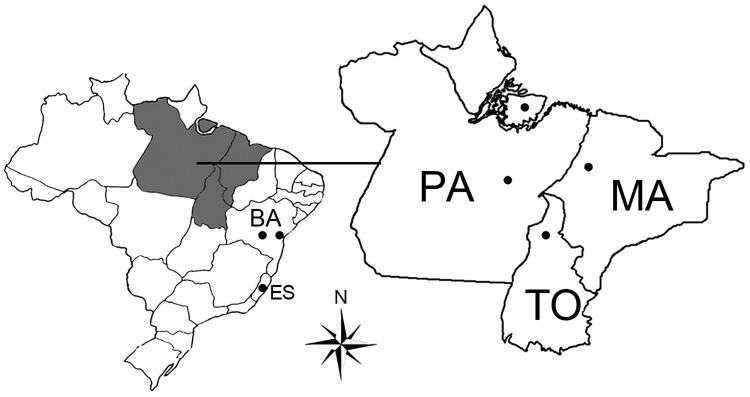
States in Brazil where serum samples were collected for study of mimivirus in mammals. Dots indicate collection sites. ES, Espírito Santos; BA, Bahia; PA, Pará; TO, Tocantins; MA, Maranhão.

We selected 321 serum samples collected from wild monkeys from the Amazon region of Brazil during 2001–2002: 91 from black howler monkeys (*Alouatta caraya*) and 230 from capuchin monkeys (*Cebus apella*). Samples were collected in an overflow area of a fauna rescue program during the construction of a hydroelectric dam in Tocantins State (Figure 1, Appendix). The monkeys had no previous contact with humans. After blood collection, the animals were released into areas selected by environmental conservation programs. We also collected serum samples from cattle (*Bos taurus*): 147 samples from Pará and Maranhão States in the Amazon region and 45 from Bahia and Espírito Santo States in the Caatinga and Mata Atlântica biomes. 

**Figure 2:**
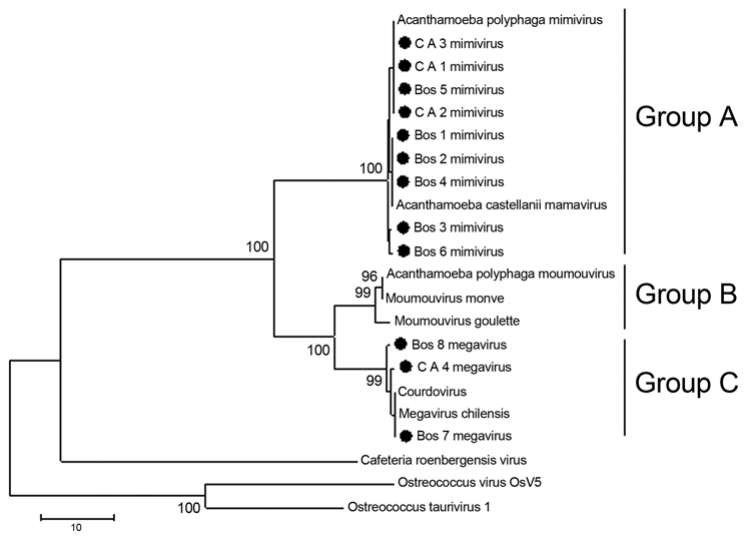
Consensus bootstrap phylogenetic neighbor-joining tree of helicase gene from nucleocytoplasmic large DNA viruses showing alignment of mimivirus and megavirus isolates obtained from *Cebus apella* (CA) and bovids (Bos) in Brazil. Tree was constructed by using MEGA version 4.1 (www.megasoftware.net) on the basis of the nucleotide sequences with 1,000 bootstrap replicates. Bootstrap values >90% are shown. Nucleotide sequences were obtained from GenBank. Scale bar indicates rate of evolution.

All samples underwent serologic and molecular testing for mimivirus (Figure 2). Because total serum volumes were low, the specimens were grouped into pools of 2–5 serum samples (20 μL for each sample) from animals belonging to the same species that were from the same collection area. A total of 210 pools were compiled (Table). Pools were tested by real-time PCR targeting the conserved helicase viral gene (primers 5′-ACCTGATCCACATCCCATAACTAA-3′ and 5′-GCCTCATCAACAAATGGTTTCT-3′). DNA extractions were performed by using phenol-chloroform-isoamyl alcohol, and DNA quality and concentration were checked by using a nanodrop spectrophotometer (Thermo Scientific, Waltham, MA, USA). PCRs were performed by using the One Step SYBr Green Master Mix (Applied Biosystems, Foster City, CA, USA), and real-time PCR quality and sensitivity parameters were adjusted, including efficiency (102.6%) and R^2^ (0.992). APMV (kindly provided by Didier Raoult, Marseille, France) was used as a positive control. The serum pools were manipulated in a laminar flow cabinet, separate from any virus samples, to avoid cross-contamination.

**Table:** Sources and test results for serum samples from wild and domestic animals analyzed for presence of mimivirus, Brazil*

Species	States where samples were collected	Total no. samples	Total no. pools	Real-time PCR, helicase gene		VN >90%, no. (%) pools
				No. (%) negative pools	No. (%) positive pools	
Black howler monkeys	Tocantins	91	21	21 (100.0)	0	0
Capuchin monkeys	Tocantins	230	106	97 (91.5)	9 (8.5)	5 (4.72)
Cattle	Pará and Maranhão	147	63	47 (74.6)	16 (25.4)	10 (15.9)
	Espírito Santo and Bahia	45	20	20 (100.0)	0	0
Total		513	210	185 (88.1)	25 (11.9)	15 (7.14)

*VN, virus neutralization test.

Of the 210 pools, 25 (11.9%) were positive for APMV (viral loads 1.4 × 10^3^ to 2.3 × 10^6^ copies/mL); 9 (4.3%) pools were capuchin monkey serum and 16 (7.6%) were bovine serum, all from the Amazon region. Mimivirus DNA was not detected in serum from black howler monkeys or cattle from Bahia and Espírito Santo States (Table). Using external primers 5′-ACCTGATCCACATCCCATAACTAAA-3′ and 5′-ATGGCGAACAATATTAAAACTAAAA-3′, we amplified a larger fragment of the helicase gene (390 bp) from all 25 positive samples; 12 positive serum pools, 4 from capuchin monkeys and 8 from cattle, were chosen for helicase gene sequencing and analysis. The helicase fragments were directly sequenced in both orientations and in triplicate (MegaBACE sequencer; GE Healthcare, Buckinghamshire, UK). The sequences were aligned with previously published sequences from GenBank by using ClustalW (www.clustal.org) and manually aligned by using MEGA software version 4.1 (www.megasoftware.net). Modeltest software (www.molecularevolution.org/software/phylogenetics/modeltest) was used determine which model of evolution was most appropriate for our analysis. 

**Figure 3:**
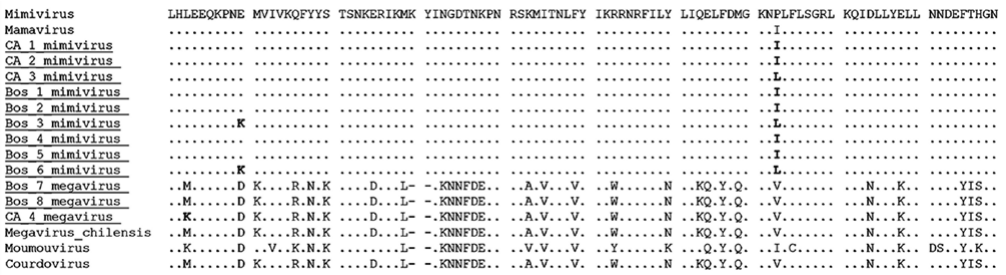
Amino acid inferred sequence of a fragment of the nucleocytoplasmic large DNA virus helicase gene (130 aa were inferred from the obtained 390-bp sample). Samples obtained in this study are underlined; boldface indicates polymorphic.

Optimal alignment of the predicted highly conserved helicase amino acid sequences showed several amino acid substitutions in the mimivirus amplicons we derived compared with other available sequences (Figure 3). Nine of the 12 sequenced amplicons showed high identity among themselves and with the APMV sequence (GenBank accession no. HQ336222). The other 3 amplicons showed a high identity with *Megavirus chilensis* (GenBank accession no. JN258408), a giant virus isolated in 2011 from seawater off the coast of Chile (*15*). A neighbor-joining phylogenetic tree constructed on the basis of the helicase gene revealed that all the amplicons we derived clustered with mimivirus isolates; however, according to the sequences alignment analysis, 3 of them clustered directly with the *Megavirus chilensis* group (Figure 2). The sequences have been deposited in GenBank.

Concomitantly with molecular analysis, the pools of samples were submitted to a virus neutralization (VN) test to detect mimivirus neutralizing antibodies. VN was used rather than ELISA because the secondary antibodies required for an ELISA for monkey species were unavailable. Before being arranged in pools, the serum samples were inactivated separately by heating at 56°C for 30 min. Inactivated samples were diluted 1:20, mixed with 10^7^ APMV particles to a final volume of 400 μL (peptone yeast glucose medium), then incubated at 37°C for 1 h to optimize virus–antibody interaction. This solution was added to 2 × 10^5^ *Acanthamoeba castellanii* cells (ATCC 30234). To improve virus–ameba contact, the adsorption step was performed while rotating for 60 min. The samples were then centrifuged at 400 × *g* for 5 min, the supernatants were discarded, and the amebas were cultivated at 32°C for 8 h in PYG medium. Afterward, the samples were titrated in *A. castellannii* cells by using the 50% tissue culture infective dose method. Antimimivirus serum produced in Balb/c mice was used as VN positive control, and bovine serum collected during previous studies by our group was used as VN negative control. The percentage of reduction was calculated, and the cutoff for positive serum was defined as 90% of the reduction in comparison with the negative control. VN results showed that 15 of the 25 PCR-positive pools contained neutralizing antibodies against mimivirus, 5 from *C. apella* monkeys and 10 from cattle (Table).

## Conclusions

We found evidence of mimivirus circulation in wild and domestic animals in the Amazon region of Brazil. Several agents of emerging infectious diseases in humans have reservoirs in wild and domestic animals, which act as a regular source for these agents. Anthropogenic disturbance of the Amazon ecosystem and increases in agricultural and livestock areas result in more contact between wildlife and rural human populations (*2*). Therefore, although mimivirus-associated pneumonia has not been studied in human patients in Brazil, surveillance of wild and domestic animals can help predict outbreaks and lead to establishment of control measures. 

Although mimiviruses are known to be present in water and soil environments, new studies are necessary to determine if these viruses are a part of a vertebrate’s normal microbiota and act as opportunistic pathogens for pneumonia and to clarify whether viruses that are associated with pneumonia have any special genetic and physiologic features. Ecologic and public health studies should be designed to evaluate the risk for infection by mimiviruses during wildlife conservation efforts and to determine whether surveillance systems can predict outbreaks by monitoring mimivirus infections in wild and domestic animals. 
